# Long term use of bevacizumab in the treatment of triple negative breast cancer with giant tumor in chest wall: A case report

**DOI:** 10.1097/MD.0000000000013410

**Published:** 2018-11-30

**Authors:** Xinyu Gui, Huiping Li, Guohong Song, Bin Shao, Hanfang Jiang

**Affiliations:** Key laboratory of Carcinogenesis and Translational Research (Ministry of Education/Beijing), Department of Breast Oncology, Peking University Cancer Hospital & Institute, Beijing, P.R. China.

**Keywords:** bevacizumab, breast cancer, chest wall metastasis, triple negative

## Abstract

**Rationale:**

Triple-negative breast cancer (TNBC) is associated with unfavorable prognosis due to lack of targeted agents. Bevacizumab, an anti-angiogenic monoclonal antibody against vascular endothelial growth factor A, has shown clinical effects in patients with TNBC.

**Patient concerns:**

We reported a 49-year-old woman presenting with a giant breast tumor.

**Diagnoses:**

Stage IV TNBC with chest wall metastasis.

**Interventions:**

The patient underwent long-term use of bevacizumab combined with chemotherapy.

**Outcomes:**

The patient was on follow-up for 46 months, a remarkable improvement of the chest wall cutaneous lesion was observed.

**Lessons:**

Bevacizumab may provide benefits for TNBC patients with chest wall metastasis.

## 1 Introduction

Breast cancer is the most common malignant disease in women worldwide.^[1]^ It is widely accepted that breast carcinoma is a heterogeneous disease in terms of histology, therapeutic response, dissemination patterns to distant sites and patient outcomes.^[2]^ Triple-negative breast cancer (TNBC) does not express clinically significant levels of the estrogen receptor (ER), progesterone receptor (PgR), and human epidermal growth factor receptor 2 (HER2).^[3]^ The clinical characteristics of TNBC include younger age at disease onset, poorer prognosis, and association with BRCA1 mutations.^[4]^ TNBC is associated with an unfavorable prognosis due to the lack of targeted agents. Breast cancer with skin involvement has a poor prognosis partly due to the limited treatment options.

Angiogenesis is a prerequisite for breast cancer growth, invasion, and metastasis.^[5]^ Bevacizumab is an anti-angiogenic monoclonal antibody against vascular endothelial growth factor (VEGF) A, which suppresses tumor growth by inhibiting neoangiogenesis. Bevacizumab combined with chemotherapy is superior to chemotherapy alone in progression-free survival and the rates of response in several large randomized trials.^[6–8]^ However, the efficacy of bevacizumab on TNBC patients with chest wall involvement has not been described before. Herein, we reported a disseminated TNBC patient with giant cutaneous metastasis and a remarkable response to long-term use of bevacizumab.

## 2 Case presentation

**Figure 1:**
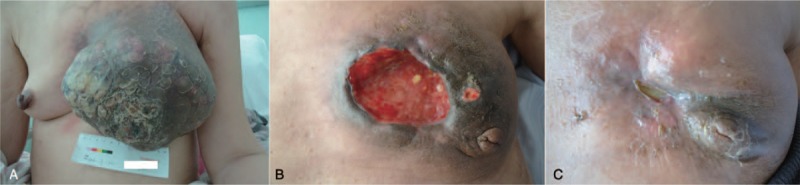
The chest wall cutaneous lesion of the patient before (A) and after (B and C) treatment.

A 48-year-old premenopausal woman was referred to local hospital for the presence of a left breast mass. Needle biopsy analysis was performed and an infiltrating ductal carcinoma with ER-negative, PgR-negative, and HER2-negative was diagnosed. The clinical examination showed giant tumor of the left breast and chest wall metastasis, together with ulcer and infection (Fig. 1A). Multiple metastases were detected in left supraclavicular fossa, bilateral axilla, anterior abdominal wall lymph node and left cervical lymph node in positron emission tomography/computed tomography. The tumor stage was cT_4_N_1_M_1_. Considering both the histologic characteristics and disease burden, chemotherapy and anti-angiogenesis therapy were performed. The treatment was initiated with 4 courses of bevacizumab 7.5 mg/kg q2w + paclitaxel 80 mg/m^2^ q3w followed by 2 courses docetaxel 35 mg/m^2^ q3w + bevacizumab 7.5 mg/kg q2w. The investigations showed a remarkable tumor regression. However, Grade 3 hematological toxicities were recorded and the treatment was stopped. Then, bevacizumab 7.5 mg/kg q2w and carboplatin 550 mg q3w was administered for 6 cycles. After that, a significant improvement of the cutaneous lesion was observed while the treatment was interrupted for the patient's poor compliance treatment due to the grade 3 leucopenia (Fig. 1B). Taking her leucopenia into consideration, 7.5 mg/kg q2w bevacizumab and cisplatin 70 mg/m^2^ q3w were administered. The skin involvement showed signs of aggression after 1 cycle. Radiotherapy was then performed to achieve local control. Xeloda 1 g bid was given for 2 weeks followed by bevacizumab 400 mg q2w and xeloda 1 g q3w from the next 1 year. Considering the patient's response to bevacizumab, 7.5 mg/kg q2w bevacizumab and gemcitabine 1000 mg/m^2^ q3w were given. A rapid improvement of the skin involvement was then observed. At the most recent follow-up (46 months from the start of treatment), a remarkable improvement of the chest wall cutaneous lesion was observed (Fig. 1C).

## 3 Discussion

Breast cancer patients can present with chest wall involvement at the time of initial diagnosis. The incidence of breast carcinoma metastasized to chest wall cutaneous is 23.9%.^[9]^ Histologically, the skin metastasis in breast cancer may be related to CCR10 and its skin-specific ligand CCL27/CTACK.^[10]^ The method most commonly used at the metastatic chest wall cutaneous involvement in breast cancer remains systemic chemotherapy. TNBC is a highly invasive and metastatic subgroup of breast cancer. Despite the striking improvement in the therapy of breast carcinoma during the last decade, TNBC remains a therapeutic challenge. TNBC is biologically aggressive, with unfavorable prognosis due to high risk of metastasis. The lack of a recognized molecular-oriented therapeutic target makes TNBC an extremely challenging medical problem.

VEGF has been indicated as the major angiogenic factor in human cancer. Increased VEGF expression is known to promote tumor growth, invasion, and metastasis.^[11]^ VEGF levels were shown to be remarkably higher in TNBC than in non-TNBC.^[12]^ Inhibiting the VEGF could reverse the enhanced migratory and invasive abilities of human TNBC cells.^[13]^ These results suggest that anti-angiogenesis may pave a new way for the treatment of TNBC.

Bevacizumab, which targets VEGF, has been investigated in breast cancer. Our previous study demonstrated that bevacizumab combined with chemotherapy significantly increased the rate of pathological response among patients with HER2 negative advanced breast cancer.^[14]^ Li et al^[15]^ recently performed a meta-analysis to investigate bevacizumab combined with chemotherapy in metastatic breast cancer and found that bevacizumab combined with chemotherapy produced a substantial improvement in progression-free survival. The E2100 trail also demonstrated that paclitaxel plus bevacizumab was well-tolerated and significantly prolonged the progression-free survival.^[7]^ Although in BEATRICE trail, which evaluated the efficacy of bevacizumab in early TNBC patients in phase III trail, showed no significant increase in overall survival.^[16]^ A further subgroup analysis of the BEATRICE study showed advantages for the addition of bevacizumab to chemotherapy alone in TNBC patients with higher level of plasma VEGFR-2. A subgroup analysis of the RIBBON-2 study indicated that the combination of bevacizumab with second-line chemotherapy might be beneficial in TNBC patients’ subset.^[6]^ Although the efficacy of bevacizumab in overall breast cancer population is unclear, our finding suggested that there might be subgroups of breast cancer population in whom bevacizumab had beneficial effects. The underlying mechanism of bevacizumab on chest wall cutaneous lesions remains unclear but several studies suggested a way in which bevacizumab might be directed toward the cutaneous lesions. Skin metastasis of breast cancer was identified to have an increased expression of androgen receptor.^[17]^ Androgen, which was demonstrated to contribute to breast cancer metastasis in rodents, had pro-angiogenesis effect and could promote the release of proangiogenic factors including VEGF.

Our finding suggested the great efficacy of the long-term use of bevacizumab combined with chemotherapy on chest wall metastasis. Further investigations are needed to investigate the mechanism of bevacizumab in TNBC patients with chest wall metastasis.

## Acknowledgment

We would like to thank Miaoning You for helping provide the Patient's clinical data for analysis.

## Author contributions

**Conceptualization:** Huiping Li.

**Data curation:** Guohong Song.

**Formal analysis:** Hanfang Jiang.

**Investigation:** Bin Shao, Hanfang Jiang.

**Writing – original draft:** Xinyu Gui.

**Writing – review & editing:** Xinyu Gui, Huiping Li, Guohong Song, Bin Shao, Hanfang Jiang.
